# Reduction Method for a Network-on-Chip Low-Level Modeling

**DOI:** 10.3390/mi16101096

**Published:** 2025-09-26

**Authors:** Evgeny V. Lezhnev, Aleksandr Y. Romanov, Dmitry V. Telpukhov, Roman A. Solovyev, Mikhail Y. Romashikhin

**Affiliations:** 1 HSE University, 101000 Moscow, Russia; elezhnev@hse.ru (E.V.L.); romashikhin.m.y@hse.ru (M.Y.R.); 2 AlphaChip LLC., 124498 Moscow, Russia; telpukhov@alphachip.ru (D.V.T.); roman.solovyev.zf@gmail.com (R.A.S.)

**Keywords:** computer-aided design (CAD), network-on-chip (NoC), RTL, HDL, modeling

## Abstract

This article explores the concept of low-level modeling of networks-on-chip (NoCs). A method for reducing the low-level NoC model by replacing the real IP blocks with a data packet generator module is proposed. This method is implemented in the low-level NoC modeling ECAD tool HDLNoCGen. This makes it possible to significantly increase the maximum number of nodes in the simulated NoC, as well as speed up the modeling and investigate the resource costs for network synthesis. A universal interface that can be used to connect new components to the network is also described. This interface has two main benefits: it reduces connection resource costs by eliminating the need to modify the connected component and shortens the time required to configure the connection interface itself. The proposed methodology of low-level NoC modeling is shown to be effective in analyzing the operation of routing algorithms of the NoC communication subsystem based on various topologies.

## 1. Introduction

The emergence of networks-on-chip (NoCs) [1] as the next stage in the development of systems-on-chip presents new challenges to computer-aided design systems (CADs) for the development of integrated circuits. The development of new tools for NoC design automation, modeling, and prototyping is required. In the classical digital system design process, the following phases can be distinguished:

- Development of technical specifications;
- Construction of high-level models in high-level languages;
- Splitting the system into functional blocks and specifying their characteristics;
- Development of behavioral models in hardware description languages (HDLs);
- Prototyping and verification of the developed system;
- Adaptation of low-level models to the requirements of the chip manufacturer;
- Estimation of the physical properties of the implemented system;
- Tape-out;
- Packaging.

The development of new approaches to the design of computer systems leads to changes in the design process itself, and the most important phase becomes the modeling phase of the developed system.

In the domain of NoC design, two distinct modeling approaches can be identified, which facilitate comprehensive research investigations. These modeling techniques are high-level and low-level modeling [2]. Consequently, these methods enable the estimation of the unique characteristics of the developed system’s operation at an early stage. In most cases, the process of data transmission in a NoC is studied in a general way, and it is impossible to take into account all the peculiarities of its hardware implementation. In high-level modeling, alterations to the description of a NoC model are generally not implemented as the model is hardware-independent. In the context of low-level modeling, where the entire system is represented in RTL form, the study of the system can be undertaken with a range of architectural modifications. Such alterations may encompass the division of a NoC into structural blocks that delineate its discrete components, modifications to the methodology of information packet exchange between NoC components, and alterations to the component descriptions themselves, among other potential modifications [3].

In the process of NoC design, a significant undertaking is the development of its communication subsystem. This subsystem (in its general form) encompasses the delineation of numerous characteristics of the NoC components. These characteristics include the topology of router connections, routing algorithm, structure of routers, and methods of controlling and arbitrating data flows in the network. The general NoC encompasses such components as computational nodes and external peripherals. However, their impact on the network operation during the initial phase can be disregarded as they do not affect the communication subsystem directly. Instead, they merely generate data that is subsequently transmitted by the communication subsystem. The interconnection between the NoC IP blocks and their communication subsystem occurs only at the level of the network routers.

Further, we define a NoC (Section 2) and review existing tools for NoC modeling, as well as describe the complexity of modeling multiprocessor systems with a large number of computing nodes. In Section 3, we propose a network reduction method and a universal interface for connecting IP blocks to routers. The study of the simulation results using different software cores and the solution proposed is described in Section 4. Finally, Section 5 contains conclusions about the model developed and the results obtained.

## 2. Background

### 2.1. Networks-on-Chip

**Figure 1:**
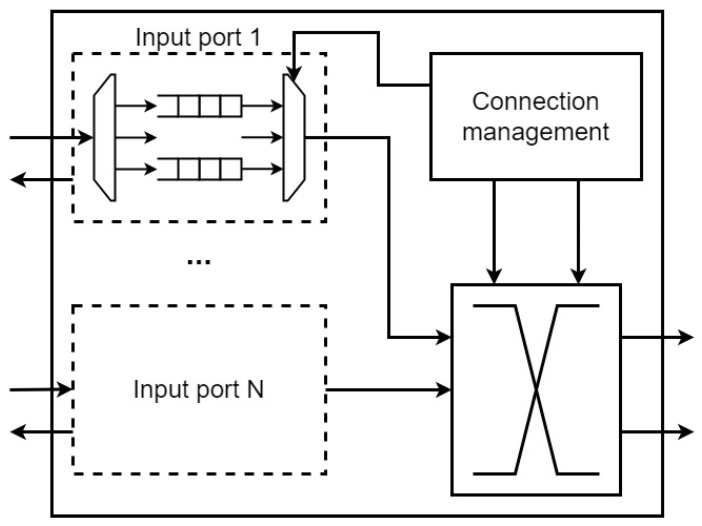
General structure of a NoC router.

A NoC is a set of computing modules on a chip with a common communication subsystem that allows them to exchange data. An IP module is often a soft-core processor, such as Nios II [4]. Every IP module is connected to a router using a communication interface. Routers are the main component of the communication subsystem of the network. The general structure of a router is shown in Figure 1.

Input and output ports (depending on the implementation) can have virtual channels [5,6]. In network operation, the required output port and additional routing information for packets are determined by the routing algorithm.

The network topology determines the way of communication between routers. There are different NoC topologies with their advantages and disadvantages [7,8]. The chosen topology directly affects the diameter and average distance of the network topology graph, determining the maximum and average delay in the packet transportation. At the same time, the network communication subsystem uses a significant amount of chip resources, which does not allow NoC modeling with a large number of computational cores.

### 2.2. Behavioral Modeling of Networks-on-Chip

The NoC design (in its general form) is characterized by the delineation of distinct characteristics. The following topics are to be addressed: the NoC topology, routing algorithm, router architecture, methods for managing and arbitrating traffic flows in a network, types of computing IP cores, and the connection structure of the components.

In order to analyze the impact of certain decisions on the performance of a designed NoC, it is necessary to carry out its comprehensive modeling. As previously indicated, two approaches are generally employed for NoC modeling: HDL and high-level languages (HLLs). While models developed in hardware description languages are inherently more accurate, they have the significant disadvantage of requiring a considerable amount of time to develop. The utilization of high-level models results in a substantial reduction in the time required for model construction; however, this reduction in time can be accompanied by a concomitant decrease in the accuracy of the results obtained.

To eliminate the disadvantage (duration) of low-level modeling, it is possible to perform co-simulation of a low-level model using FPGA when a part of the model is loaded into the FPGA, and prototyping is performed there while the results are already processed on the workstation, where the other part of the model is executed. Another way to accelerate modeling is the joint execution of a code written in HLL and HDL code by using special tools VPI/PLI, DPI [9], and others. Also, at this phase, means of translation of high-level models from such software as MATLAB into low-level representation can be used [10]. Such tools are becoming more and more popular and even received a separate name—High-Level Synthesis (HLS) tools [11,12]. But at present, they are still underdeveloped and have many limitations.

There are solutions that can speed up the NoC modeling process. But only quite a few among them are specialized high-level models (e.g., BookSim [13,14], VNoC [15], Noxim [16], and NoCTweak [17] et al. [18,19,20]); fewer are low-level models (e.g., [2,21,22,23,24]), but all of them are designed for certain narrow tasks and poorly modifiable. Also, various automation tools for NoC modeling and other software were developed by various authors [11,25,26].

Work [25] presents the tools for modeling embedded processors—Liberty Simulation Environment (LSE). The proposed solutions are based on the network architecture development system, Mescal [27]. LSE is a tool that can automatically map the parallel and structural specification of the microarchitecture of RISC processors to the model structure. Given that LSE automatically maps microarchitecture specifications into a sequential program, LSE’s microarchitecture resembles the hardware it models. Consequently, only minor alterations in the specification are required to simulate minor alterations in the microarchitecture. Furthermore, LSE was conceived to emulate specialized hardware that is prevalent in specialized processors but less frequently employed in general-purpose processors. The advantages offered by LSE enable developers to prioritize learning microarchitecture, as opposed to dedicating resources to the management of an inherently intricate and opaque sequential model. In LSE, the user defines a NoC model by describing instances of architectural components (called modules) and their inter-port connections. Each module is approximately analogous to a hardware block and features parallel execution semantics similar to those of real hardware blocks. This characteristic serves to simplify the specification and enhance its precision. The LSE specification enables the user to customize and extend modules through the module extension mechanism. Users have the option of utilizing preexisting modules from the LSE library or creating their own. The task of describing a comprehensive microarchitecture for modeling is frequently arduous. The development of novel microarchitectures for a specific subject area frequently entails the modeling of components of the microarchitecture, while eschewing the consideration of superfluous details. LSE provides this type of partial specification by assigning default values to unconnected ports. It is reasonable to expect that modules will behave in an acceptable manner in the event that some ports remain unconnected. A complete description of the network architecture can be developed incrementally step by step.

HLS tools are reviewed in ref. [11]. A number of criteria to choose certain HLS tools are given: programming languages (to implement synthesis tools) themselves, complexity of working with them, possibility of adding new components, and feasibility of verification of the system under study, ability to generate an RTL description of the system, etc. The paper explores a number of problems with HLS tools that make their use not always appropriate. For example, there is often a lack of standardization when entering a description of the system under study. This leads to developers requiring additional training in designing with a particular tool. In all tools (even the best ones), exploration and optimization capabilities can be extended (improvement of data localization and reduction in bandwidth requirements of various network components). Exploration of the designed NoC and optimization of its architecture frequently necessitate modification of the source code of its model, thereby disrupting the separation between the functional components of the model. It is important to note that certain tools are designed for a single application domain; for example, these tools are exclusively used for describing data flows or controlling logic. This represents a substantial disadvantage, as developers are compelled to utilize numerous tools and manually adjust the data they generate to align with their specific tasks.

In ref. [26], various NoC models are reviewed. It is shown that some of them are commercial products that cannot be freely used. Half of the models studied are not synthesizable in hardware, which does not allow their full use for the NoC analysis. At the same time, it is noted that the results of modeling with the use of different models are not consistent in terms of data representation formats.

As a result of a review of publications on using modeling tools and automating various modeling processes, it can be concluded that there is no single universal tool capable to allow the implementation of the NoC end-to-end design principle, where different models and tools can be used at different phases of design flow, and their compatibility through the implementation of universal interfaces and representation of input and output modeling data are ensured.

The objective of modeling is to estimate the primary characteristics of the system under development, including consumed chip resources, throughput, power consumption, and time delays. Depending on the means and the stage of NoC design modeling, there are several levels of modeling abstraction that can be distinguished:

1. Analytical level. It is the process of deriving, analyzing, and approximating analytical formulae that describe the processes occurring in a NoC. An example of such a model is formulated as an expression that represents the solution to the problem of minimizing the energy expenditure of communication between network nodes [19]. Another example is presented in [28], where the dependence of the speed of parallel data processing on the NoC parameters is expressed in a mathematical formula. The influence of delays in data transmission as the network dimension increases is analyzed.
2. High-level. In the early stages of design, high-level modeling is typically conducted using HLL [2,14,17,29,30].
3. Low-level. Low-level modeling involves studying the use of chip resources and the power consumption of the designed system at the stage of its prototype described in HDL [2,21,31].
4. Mixed level. The term “mixed level” is employed to denote the process of network traffic modeling and analysis. This involves the examination of various parameters, including bandwidth, packet transmission delays, and the efficacy of routing algorithms within a NoC [32,33,34,35].

A salient feature that distinguishes high-level models is their inability to synthesize into a real NoC. It has been demonstrated that low-level models are inherently devoid of this disadvantage and consequently exhibit enhanced accuracy, as they effectively serve as a NoC prototype. The simulation is characterized by its extended duration, a consequence of its cycle-accurate [36] nature and the employment of event-driven simulation tools, such as ModelSim [37]. This approach enables the generation of an accurate, highly parameterized NoC model (e.g., Netmaker [5]). The primary disadvantage of this approach is that the modeling process is excessively time-consuming. The utilization of simplified and low-level NoC models (e.g., NoCSimp [38]) facilitates the reduction in simulation time by an order. A potentially fruitful approach to examining the utilization of chip resources would involve prototyping it on an FPGA development board. This approach allows estimating the same parameters much faster compared to low-level modeling, while there is an opportunity to verify the performance of the system.

High-level models hypothesize which components with which parameters can be used in a NoC design to obtain the required NoC performance characteristics. Such modeling does not break down the system into functional blocks. Low-level models (due to their organization) provide precise characteristics of the network operation and its structure with reference to the hardware implementation.

According to [39], the maximum simulation speed with ModelSim is $3.2\times 10^{3}$ cycles/s, SystemC—$20\times 10^{3}$ cycles/s, while the FPGA chip operates with an operating frequency from 50 MHz ($50\times 10^{6}$ cycles/s). The development of low-level modeling is the implementation of the network on the FPGA chip (prototyping), where the analysis is performed in real time at the hardware level [35,39,40]. This approach is time-consuming for design development and requires special equipment; it is used in the late phases of design.

It should be noted that the development of low-level models involves the need to create a large amount of single-type code to test various characteristics of NoC components, which is resource-intensive both in terms of time and in detecting errors in the NoC description. At the same time, there are actually no tools for automating model development. The development of such tools allows for reducing the resource costs and total time for model development and obtaining modeling results.

Thus, high-level NoC models are not sufficient to evaluate the benefits of a particular NoC configuration. High-level models allow fast selection of optimal network parameters, but they cannot be used to test individual components or develop a complete network. At the same time, using low-level models is applicable to study individual components of the network and generate NoC prototypes for testing on FPGA. The model should be tolerant to change: it should support different topologies and types of routers, be extensible but not cumbersome to enable NoC prototyping with a large number of nodes on FPGA chips at an affordable cost, and be fast enough to run the model using event-driven modeling tools.

### 2.3. Survey of Low-Level NoC Models

While there are many more high-level models developed, there are also a fair number of low-level models. However, most projects investigate NoC elements under different conditions. Usually, the object of study of a low-level model is network topology, network dimensionality, router structure, routing algorithm, and other parameters. Consider some well-known low-level NoC models in the context of their purpose and parameters.

In the Verilog-based-NoC-simulator project [22], the structure of a router that is connected using a 5×5 mesh topology is tested. The network is used to transmit the traffic of different natures and to track (by means of modeling) the transmission delay of each packet. The structure of the applied router differs from the classical one, which uses a single arbiter for routing. Instead, each output module contains its own specialized arbiter. The coordination between the arbiters is achieved by means of a special bus. As in many other mesh topology designs, this router uses the XY routing algorithm [3]. The packet is first transmitted to the next routers along the X coordinate and then along the Y coordinate to the destination node. Moving along the axes in strict order prevents the formation of closed loops in the network traffic if certain turns are prohibited in the network [3]. Closed loops put routers in a perpetual waiting state, where each router waits for its neighbor’s buffer to be released.

Another variant of the NoC model is the LAG project [23]. It investigates the link aggregation by which network bandwidth can be improved. It is proposed to add additional physical links between routers due to which makes it possible to transmit multiple flits between routers simultaneously. Here, each packet is divided into flits, which follow each other in strict order along a given route. Flits can also be transmitted in an arbitrary order using multiple routes. In this case, the receiver has to reconstruct the queue of flits to read the packet correctly. Such correction requires significant time and chip resources; hence, transmitting flits in an arbitrary order is not always preferable [3]. On the other hand, the orderly transportation of flits increases the probability of head-of-column blocking (HOLB), resulting in a possible lowering of the network bandwidth [41]. While the head of the column is waiting for permission to move further, consecutive flits are forced to wait. To solve this problem, some researchers use virtual channels [41]. This approach reduces the probability of HOLB several times but does not eliminate it completely. The authors of the project suggest that the probability of HOLB can be further reduced by replacing virtual channels with additional physical links [42]. A mesh network with 4 × 4 dimensionality, where each router is connected to its neighbors by two physical links, is designed.

In the PageRank-Sort model, the network used to implement the PageRank algorithm is used [24]. Such an algorithm belongs to the class of link ranking; it sorts web pages by authority. The authority of a page (a numerical value) is determined by the links to other pages. The more links a page has, the higher its authority. Moreover, each link contributes a weight equal to the authority of the parent page. The equation determining the authority of page u is given below (1):

(1) $$R\left(u\right)=\left(1-d\right)+d\cdot \sum \frac{R\left(v\right)}{N\left(v\right)}.$$

The parameter d corresponds to the total number of pages. The values R(v) and N(v) represent the authority and total number of links of a linked page.

The links between pages can be described by a graph. However (if the graph is large), sorting may take a significant amount of time, which will adversely affect the performance of the search engine. The project proposes running the sorting on four processors connected to each other using a NoC. The topology of the developed network is circular, and a processor handling a quarter of the graph is attached to each router. The processors can be classically linked by a common bus, but the project uses a NoC to improve performance.

The network routers are implemented in the standard way: inputs are fed into FIFO buffers, which in turn are read by the arbiter. The arbiter then routes the flits to the outputs using the built-in switch.

The project code is synthesizable but does not support parameterization of network dimensionality. The code is strictly implemented under four routers (including the routing algorithm) and does not contain parameters to describe the network dimensionality.

A NoC is often developed for multiprocessor systems where a program runs on multiple processors. The processors individually perform part of a common task and communicate with a NoC when needed. The MNOC_3rd project is developed for a different purpose: multiplexing [43]. The idea of the project is as follows:

1. There are a large number of sensors.
2. Each sensor randomly generates packets with the information.
3. Some packets have a high priority. They must be processed out of queue.
4. Only one processor handles the packets.
5. Incoming packets must be routed to the processor one at a time.

To solve this problem, the project proposes to use a NoC whose topology corresponds to the “BFT” (butterfly fat tree). The packets are gradually directed from the nodes of the tree (where the sensors are located) to the root, where the processor is located. The “CPU” block represents the processor. Blocks “i1”, “i2”, and “i3” represent the three types of a router.

Three differently structured routers are used to build the tree. Router “i1” contains one input and one output, router “i2” contains two inputs and one output, and router “i3” contains three inputs and one output. The routers contain two simple virtual channels: one for the normal packets and one for priority packets (which need to be delivered as fast as possible). The routers determine the priority of a packet by the header that is in each flit.

In work [44], the authors proposed the use of NoC to reduce the design time of specialized SoCs by utilizing special feeder-collector blocks for data transmission and preprocessing between computational blocks and the NoC. It is shown that this approach allows the implementation of DSP algorithms, demonstrated through the example of MMSE equalizer implementation.

In work [45], the authors provided a method for NoC design using initial high-level modeling of an abstract network model and its components, followed by their transformation into a low-level model in HDL. As an example, the development of a NoC switch for use in a software-defined radio system (SDR) is demonstrated. The high-level design of the switch was carried out in the OMNet++ program. The low-level model is implemented as a finite state machine.

The above review of low-level NoC models is not exhaustive, but clearly demonstrates that each of the models discussed above is designed to investigate different network characteristics. They describe a NoC with different structures, set of components, and input parameters, and generate different output data. This all makes it impossible to harmonize the simulation data from different models with each other, which increases the effort required to analyze the results. The structure of the considered models is highly coupled, which leads either to the lack of possibility to modify such models or to the necessity to completely change their code so that new features can be added and new NoC parameters be investigated. Thus, most low-level NoC models can be considered as special cases; there is no universal approach to low-level modeling, let alone automation tools for low-level NoC modeling.

### 2.4. Very Large Scale Multiprocessors

Development and prototyping of multiprocessor systems built on the NoCs is an urgent task. Large NoCs with more than 100 nodes [46,47,48] are difficult (and in most cases impossible) to place not only on the FPGA but also on the ASIC. This is due not only to the limited number of logical elements in the FPGA chips but also to the lack of the ability to design such a size of chips in development environments. To bypass this limitation, multi-FPGA complexes are used, but their use greatly complicates the development. This is due to the implementation of high-speed communication channels between the FPGA [49] and their implementation in the NoC project, as well as the need to synchronize all FPGAs included in the prototyping complex [50]. In ref. [51] the authors developed a NoC that uses statically scheduled traffic to transmit data. For a network of nine nodes, the communication subsystem alone takes up more than 15,000 logical elements. Separately, one core takes up more than 9400 logical elements. Although the full size of the network is not specified in the text of the work, it can be assumed that it takes up approximately 100,000 logical elements, which is already more than the available resources for medium-sized FPGA chips. At the same time, a small-sized network with relatively small processor cores is discussed. In ref. [52] the authors propose a methodology for designing multi-core processors with 2400 cores based on FPGAs. They prototyped such a system on a multi-FPGA complex, which required splitting the entire multiprocessor system into 100 separate partitions loaded into separate FPGAs within the multi-FPGA complex. As a result, the entire multi-core processor occupied more than 80% of the multi-FPGA complex.

Thus, designing and prototyping modern multiprocessor systems with hundreds of computing units on the NoCs is a complex, resource-intensive task. The use of multi-FPGA complexes for its solution is not always possible since it introduces inaccuracies into the operating characteristics of the original system. Therefore, it is necessary to use approaches based on separate modeling of NoC components and functional replacement of resource-intensive elements in order to reduce the consumption of hardware resources for the modeling.

## 3. Improving the NoC Low-Level Modeling

### 3.1. Reduction Method for a Low-Level NoC Model

Using real IP blocks to generate data for NoC operation, networks with a small number of nodes can be investigated. At the same time, there are already chips representing NoCs with hundreds [53] and even thousands of nodes [54,55]. The study of such NoCs is an important scientific task, and while high-level models allow this [56] (although facing the problem of computational complexity [2]), most low-level models are usually limited to tens [5,57,58,59], at most, to hundreds of nodes [38].

On the other hand, although there are currently FPGA chips with on-chip integration of logic blocks in excess of tens of millions, even these cannot accommodate a NoC with a really large number of nodes. Table 1 and Table 2 show the characteristics of the consumed chip resources by the processor cores schoolMIPS [40,60], schoolRISCV [61], Nios II [62], and SRC1 [63], as well as a theoretical evaluation of the possibility of realizing a NoC based on these cores on an FPGA chip. The evaluation considers the fact that usually the communication subsystem occupies up to 40% of the chip resources.

Nios II and SCR1 cores are indicative here since, among the considered ones, they are the most suitable for creating a NoC. The other cores can be used only for a limited set of tasks due to their simplicity.

The solution to the problem of low-level modeling of a NoC with a large number of nodes is the method of model reduction. This method is implemented in the HDLNoCGen ECAD tool for low-level NoC modeling [64]. The packet generator module is responsible for handling this task. It has been developed for the purpose of generating test data packets, the objective of which is to simulate the operation of the communication subsystem.

**Figure 2:**
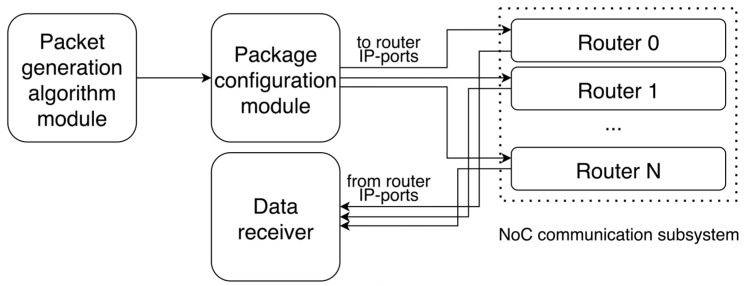
Structure of a packet generator module.

The general structure of the packet generator module is shown in Figure 2.

The packet generator module consists of several blocks. The generation algorithm block implements the type of packet generation selected by the developer. By default, this block contains only a template for generating the output vector with packets. The main part of the algorithm is implemented by the developer in traffic generation and the data packet structure itself. As a result, a vector with packets for all the routers is formed. The formed vector is transferred to the packet configuration block. In it, the original vector is divided into packets separately for each router. The prepared packets are transferred to the IP ports of the routers through which, in the case of using real computing IP blocks, they are connected to the routers.

After routing the packets by the routers, the results are collected in the data collector block. It allows evaluating the correctness of the packet transmission by the routing algorithm.

**Figure 3:**
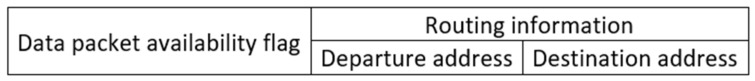
Structure of a packet generated by the packet generator model reduction module.

The developed low-level model employs a simplified packet structure as illustrated in Figure 3.

Useful data in a packet occupies only one bit (the packet presence flag). This is sufficient for testing NoC operation. The packet generator module allows customizing the length of the generated packet, but this will increase the number of resources consumed. The rest of the packet contains auxiliary information for the routing algorithm. The size of the auxiliary data depends on the routing algorithm. The pair exchange algorithm from ref. [65] is used to test the operation of the reduced model.

The packet generator module allows checking the correct operation of the NoC communication subsystem in terms of data transmission from the source node to the receiver node, but it does not simulate a real IP block completely. Given separate modeling, the functionality of the module is sufficient to check the communication subsystem between the nodes and processor cores, as well as check its connection with the processor core.

We use a DE1-SoC development board with a middle-grade FPGA chip [63] for testing the operation of the reduced model. The IP block (implementing schoolMIPS [60] soft-core processors) is connected to the NoC communication subsystem. The results of the NoC operation are displayed on the periphery of the debug board via the Data receiver module.

**Figure 4:**
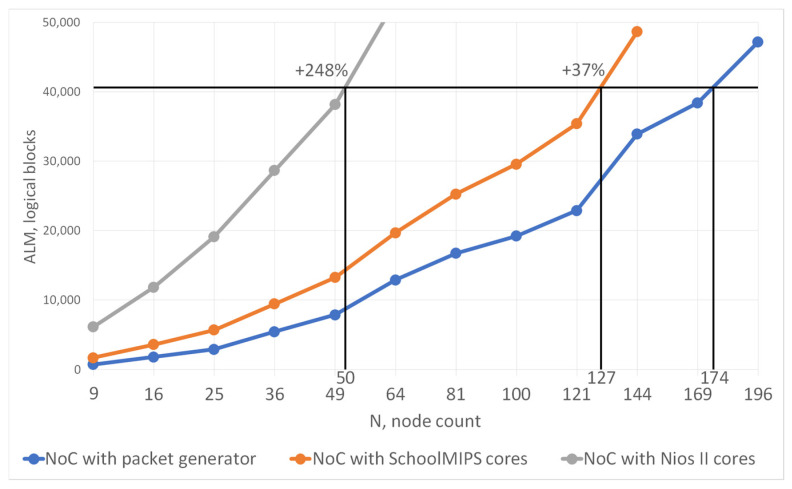
Logical resources of the FPGA chip occupied by a NoC using the real computational cores (depending on the network size).

An interface between soft-core processors and the communication subsystem is implemented to harmonize the data and ensure the required format. The main job of the interface is to convert data. Figure 4 shows a graph of the dependence of the use of logical resources of the DE1-SoC chip on the number of network nodes based on schoolMIPS and Nios II cores. This graph was obtained after modeling and prototyping the NoC on a development board.

The horizontal black line shows the number of logical blocks of the development board. As shown, the number of nodes in the network and the number of cores managed to be placed on the chip are less than specified in the table. And in general, a NoC reaching only about 50 nodes turns out to be quite small. Thus, the DE1-SoC FPGA development board allows for the implementation of a NoC with 50 Nios II nodes and 127 schoolMIPS nodes. Using real IP blocks to prototype a NoC with a large number of nodes is challenging.

The blue line is a graph of the dependence of the use of resources by the reduced model, depending on the size of the network. With the same limitation on logical resources, it is possible to place a much larger number of nodes on the chip. The increase compared to the network based on schoolMIPS cores is only 37%, but this means that the increase compared to the network based on Nios II cores is almost 2.5 times. The reduced model on the DE1-SoC development board, with not the most resourceful FPGA chip, allows analyzing a NoC with 200 nodes. The method of reducing computing blocks proposed in the work is universal and can be used in a NoC with any number of nodes.

The proposed approach has one drawback: the reduced model developed has several additional modules in its structure. They are not architectural components of the communication subsystem or NoC as a whole. However, they do participate in modeling and make distortions in the obtained results. Consider how significant these distortions are.

**Figure 5:**
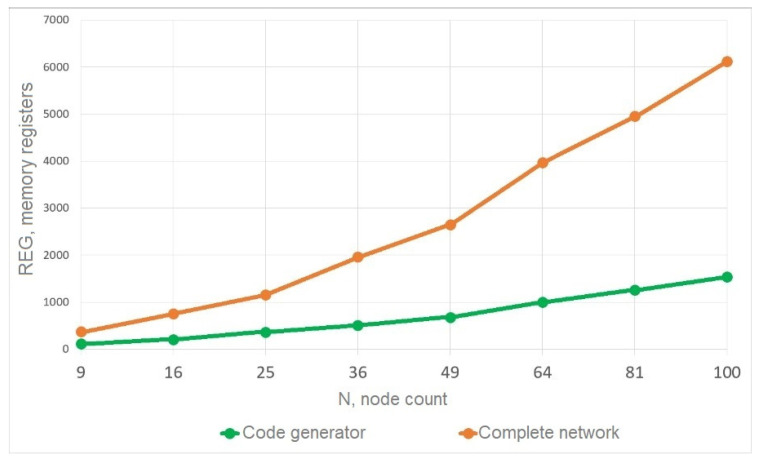
Dependence of FPGA REG usage for auxiliary modules on the number of nodes in a NoC.

**Figure 6:**
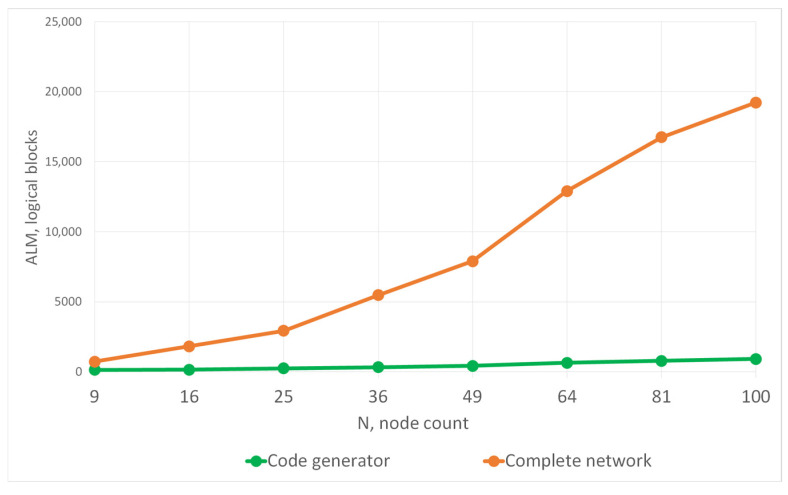
Dependence of FPGA ALM usage for the whole network and auxiliary modules on the number of nodes in a NoC.

The packet generator module’s structure is constant and directly proportional to the parameters of the NoC communication subsystems. Its basis is a vector of dimension L=N·BIT, where N is the number of nodes, and BIT is the length of the packet (or flit) generated by the generation algorithm block. This vector is then split into N vectors of length BIT, which are sent to the routers. For network testing, a “hot potato” traffic generation algorithm was implemented, which consists of one packet being transmitted randomly between nodes in the network from one to another. Modeling of the packet generator module was carried out separately from the network, as well as within the network for a different number of nodes, which altered the packet length by changing the required number of bits for storing routing information. Based on the simulations, data were obtained on the occupied resources of the packet generator module as part of the network and separately from it. These data are shown in Figure 5 and Figure 6. The green color shows the resources occupied by the packet generator module separately from the network; the orange line demonstrates the resources occupied when considering the packet generator module to be part of the reduced network.

Based on these data, formulas were obtained for calculating the chip resources occupied by the packet generator module.

For the FPGA chip registers, the formula was calculated accurately and coincides with the simulation results:

(2) $$U_{reg}=N\cdot BIT+2\cdot BIT+14.$$

The first term is the packets created by the generator module for the routers, and the second and third terms are the registers for the operation of the generation algorithm.

The formula for estimating redundancy in logical blocks (ALMs) is as follows:

(3) $$U_{alm}=0.0006\cdot N^{2}+8.546\cdot N+25.101$$

This formula is expressed by the second-degree polynomial function and is obtained approximately with the reliability coefficient $R^{2}=0.991$ by the method of polynomial interpolation of the simulation data presented in Figure 6.

For networks with fewer than 10 nodes, auxiliary modules occupy a significant portion of the total amount of resources consumed by the network (more than 60% of registers and more than 40% of logical elements). However, with the increase in the number of nodes up to 100, auxiliary modules already occupy only 17% of registers and 7% of logical elements.

The estimation using Formula (2) for memory registers is exactly the same as the theoretically calculated values. The estimation of logical resources is expressed by the second-degree polynomial function (3) with the reliability coefficient $R^{2}=0.991$. The results clearly show that the packet generator module uses a negligible number of resources compared to the communication subsystem. For a larger number of nodes, this redundancy can be neglected.

Using Formulas (2) and (3), one can calculate the introduced redundancy for a reduced NoC with any number of nodes.

**Figure 7:**
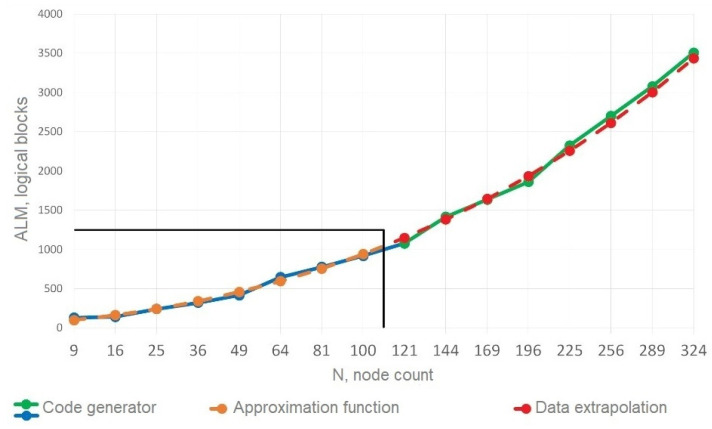
Dependence of extrapolated FPGA ALM usage for the whole network and auxiliary modules on the number of nodes in a NoC.

The estimation formulas used by non-architectural components of the NoC resources were obtained. These formulas were used to calculate the resources used for more nodes using the data extrapolation method (Figure 7).

The data obtained from the polynomial function for a NoC with the number of nodes from 121 to 324 is close to the experimentally obtained data.

The assessment of the introduced redundancy by logical resources can be estimated using the polynomial Formula (3). The size of the packet generator module does not depend on the routing algorithm used. In this case, the dependence is only on the number of nodes in the network.

The polynomial formula (the orange graph) was calculated based on the data of the blue graph located in the lower left part of the figure, which represents experimentally obtained data. Further extrapolating the data, a red graph was obtained, and the resource consumption was calculated separately (the green graph in the upper right part of the figure).

The discrepancy between practical results and extrapolation does not exceed 3% and consists of some features of the change in the length of the packet size.

### 3.2. Universal Interface for Connecting NoC Components to the Communication Subsystem

**Figure 8:**
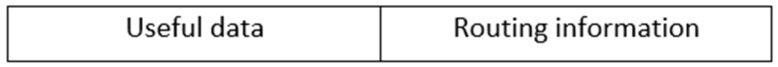
General packet structure transmitted between the IP blocks in a NoC.

To fully estimate the performance of the NoC communication subsystem, it is necessary to ensure that it correctly transmits packets from the source node to the receiver node. To do this, it is necessary to connect the IP blocks that will be engaged in the generation of data packets. For the correct operation of the communication subsystem, the transmitted data must be formed in a certain way in the form of data packets. A data packet is characterized by its size and the presence of auxiliary information for the routing algorithm. The general structure of the data packet for the operation of the NoC communication subsystem is shown in Figure 8.

The IP blocks are independent components that are not originally adapted to work in a NoC. When connecting such components, it is necessary to agree on the data format required for transmission by the NoC communication subsystem and the data format capable of receiving and sending the IP block connected. One of the options for solving the data matching problem is to modify the plug-in component to form the required data package. This solution is not universal and is only suitable when the developer has a description of the plug-in component. In addition, in this case, it is necessary to change the plug-in component itself, which will make it impossible to use it for other purposes.

**Figure 9:**
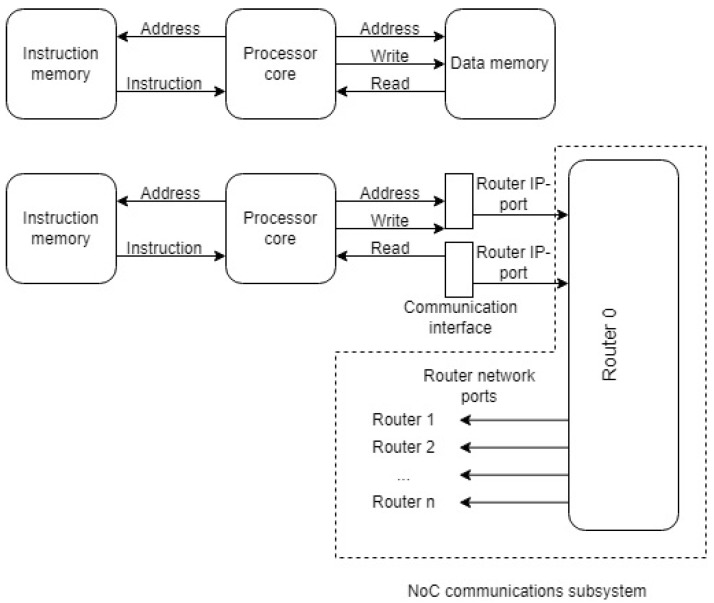
Connecting the schoolMIPS core to the NoC communication subsystem.

The developed NoC model can connect external IP blocks to the communication subsystem to check the operation of the network as a whole. The interface was not changed. Instead, a communication interface module was implemented in such a manner to negotiate the data format. The communication interface is implemented as a separate single module. The developer can configure it to connect various IP blocks. Using the example of connecting the schoolMIPS core [60] makes it possible to demonstrate the connection of an IP block to the NoC communication subsystem. The structure of its connection through the communication interface is shown in Figure 9.

At the top of the figure, there is an original structure of the schoolMIPS core. It consists of the core of the soft-processor itself, the memory containing the program instructions, and the data memory. For the correct operation of the soft-processor in the NoC communication subsystem, a communication interface is added between the processor core and the router. Structurally, it is a pair of buffers, the converters, which (in the first case) form a packet of the required length from the data from the soft-core processor and add auxiliary information to it for the operation of the algorithm, and (in the second case) remove information from the packet received from the communication subsystem for the operation of the algorithm and save the required number of packets into which the original data were divided.

Thus, to add a new component to the communication subsystem, there is no need to change it. One just needs to configure the communication interface, specifying the size of the input data packet from the plug-in component, as well as its structure.

The universal interface developed for connecting new NoC components to its communication subsystem made it possible to estimate resources consumed by a NoC based on various IP blocks. The proposed method of connecting new components to the network allows reducing resource costs by eliminating the need to modify the connected component, and also shortens the time for setting up the connection interface itself.

## 4. Discussion

All the solutions proposed, as well as methods and techniques combined in a single low-level model, were used in various studies of different aspects of NoC functioning.

### 4.1. Research and Comparison of Routing Algorithms for Various NoC Topologies

Since modeling can now be performed separately for the communication subsystem, it is much easier to implement new topologies at the HDL model level. The topology can be specified either by its type or by an incidence table, after which the communication subsystem will be synthesized for a specific case of connections between network nodes. Also, since routers have become much simpler at the level of the communication subsystem, and IP cores can be replaced by traffic generators, it is now much easier to explore new routing algorithms in a NoC, as well as different traffic distribution profiles in a network. Thus, in [66], specialized models of the NoC communication subsystem were obtained, with the help of which it is possible to model a NoC with 100 nodes, so that obtaining results increases up to 10 times. In [67], a new low-level model to test the developed routing algorithm is used.

### 4.2. Research and Comparison of the Results of Synthesis of Various NoC Configurations

Since (as shown above) it is possible to estimate the cost of resources for a NoC as the sum of the costs for the communication subsystem and IP core separately, this makes it possible to estimate the cost of chip resources for large NoCs. Thus, in refs. [68,69], in order to prove the effectiveness of the proposed routing algorithms in a NoC, large-sized communication subsystems of a NoC were synthesized, and the obtained chip resource utilization values were compared.

The developed methodology for low-level modeling NoC tools significantly reduced resource costs for creating special parameterized low-level NoC models, as well as the time for their preparation and obtaining simulation results. The testing conducted proves the applicability of the proposed solutions and demonstrates the reliability and reproducibility of the results obtained.

## 5. Conclusions

Thus, we propose a new method for reducing the low-level NoC model by replacing real IP blocks with a data packet generator module, which allows (with the same result as when using real IP blocks) to simulate a NoC with a large number of nodes reaching 200. This makes it possible to increase the size of the network under study by up to 2.5 times compared to a NoC based on real IP blocks when prototyping a NoC on the DE1-SoC FPGA board by replacing the NIOS II processor cores with a packet generator.

To connect new NoC components to its communication subsystem, a universal interface was developed to analyze the resource costs of a NoC based on various IP blocks. The proposed method of connecting new components to the network allows reducing resource costs for connection by eliminating the need to modify the connected component, and also shortens the time for setting up the connection interface itself.

The given examples of approbation of the NoC modeling technique proposed, as well as the low-level NoC model (developed on its basis in the tasks of analyzing the operation of routing algorithms of the NoC communication subsystem based on various topologies), demonstrate its high efficiency.

## Figures and Tables

**Table 1:** FPGA chip resources occupied by soft-core processors.

Soft-Core Processor	ALM	REG
schoolRISCV	99	768
schoolMIPS	137	768
Nios II	751	8192
SCR1	4464	4059

**Table 2:** Maximum theoretically achievable number of nodes in a NoC with connected soft-core processors.

Soft-Core Processor	schoolRISCV	schoolMIPS	Nios II	SCR1
CYCLONE IV EP4CGX150DF31IAD, Altera, San Jose, CA, USA	363	264	47	8
CYCLONE V 5CSEMA5F31C6, Altera, San Jose, CA, USA	252	183	33	5
MAX 10 10M50DAF484C7G, Altera, San Jose, CA, USA	120	87	15	2

## Data Availability

The data presented in this study are openly available in https://github.com/evgenii-lezhnev/HDLNoCGen (accessed on 24 July 2025).
